# Supplementary material for: Diagnosis of SARS-CoV-2 during the Pandemic by Multiplex RT-rPCR hCoV Test: Future Perspectives
Source: Pathogens. 2022 Nov 18;11(11):1378. doi: 10.3390/pathogens11111378 (PMC9694306; doi:10.3390/pathogens11111378)
Supplement: Supplementary file 1 [file pathogens-11-01378-s001.zip › Table S1.pdf]

Table S1: Comparison among the results of the GSD NovaType II SARS-CoV-2/GSD NovaType III SARS-CoV-2 kits versus the results of NGS. The 835 positive cases of SARS-CoV-2 detected by the MPL RT-rPCR were detected by confirmatory test and sequencing by NGS, too. From these results, we can conclude that the MPL RT-rPCR is sensitive to detect the SARS-CoV-2 directly from the samples regardless of the different variants.

GSD RT PCR	Pangolin Lineage	Nextstrain clade	No of detected case	GSD RT PCR	Pangolin Lineage	Nextstrain clade	No of detected case
DELTA	AY.102	Delta, AY	24	DELTA	AY.66	Delta, AY	2
	AY.103	Delta, AY	2		AY.68	Delta, AY	3
	AY.111	Delta, AY	1		AY.7.2	Delta, AY	14
	AY.116	Delta, AY	7		AY.71	Delta, AY	3
	AY.120	Delta, AY	6		AY.72	Delta, AY	4
	AY.120.2	Delta, AY	1		AY.75	Delta, AY	2
	AY.121	Delta, AY	3		AY.77	Delta, AY	1
	AY.122	Delta, AY	46		AY.89	Delta, AY	2
	AY.122.1	Delta, AY	1		AY.9.1	Delta, AY	2
	AY.124	Delta, AY	4		AY.92	Delta, AY	11
	AY.125	Delta, AY	25		AY.96	Delta, AY	1
	AY.19	Delta, AY	3		AY.98	Delta, AY	1
	AY.22, .23	Delta, AY	2		AY.98.1	Delta, AY	19
	AY.25	Delta, AY	1		B.1.1.7	Alpha, V1	1
	AY.33	Delta, AY	5		B.1.525	Eta	1
	AY.34	Delta, AY	27		B.1.617.2	Delta	60
	AY.36	Delta, AY	2		BA.1	Omicron	1
	AY.37	Delta, AY	2	ALPHA	AY.4	Delta, AY	1
	AY.39	Delta, AY	2		B.1.1.7	Alpha, V1	38
	AY.4	Delta, AY	44		B.1.375	Beta?	1
	AY.4.2.2	Delta, AY	1		Q1	Alpha, Q1	9
	AY.4.2.3	Delta, AY	2		Q4	Alpha, Q4	1
	AY.4.5	Delta, AY	2	GAMMA	AY.117	Delta, AY	1
	AY.40	Delta, AY	2		AY.125	Delta, AY	1
	AY.42	Delta, AY	30		B.1.1.7	Alpha, V1	1
	AY.43	Delta, AY	169		P.1	Gamma	1
	AY.44	Delta, AY	3		P.1.12	Gamma	3
	AY.46.6	Delta, AY	31	OMICRON	B.1.1.529	Omicron	1
	AY.5	Delta, AY	17		BA.1	Omicron	109
	AY.53	Delta, AY	2		BA.1.1	Omicron	39
	AY.58	Delta, AY	2		BA.2	Omicron	1
	AY.6	Delta, AY	1	OTHER	B.1.1.523	Omicron?	2
	AY.61	Delta, AY	27		C.37	Lamda	1
